# Asian Culturally Specific Predictors in a Large-Scale Land Use Regression Model to Predict Spatial-Temporal Variability of Ozone Concentration

**DOI:** 10.3390/ijerph16071300

**Published:** 2019-04-11

**Authors:** Chin-Yu Hsu, Jhao-Yi Wu, Yu-Cheng Chen, Nai-Tzu Chen, Mu-Jean Chen, Wen-Chi Pan, Shih-Chun Candice Lung, Yue Leon Guo, Chih-Da Wu

**Affiliations:** 1 National Institute of Environmental Health Sciences, National Health Research Institutes, Miaoli 35053, Taiwan; gracecyhsu@nhri.org.tw (C.-Y.H.); yucheng@nhri.org.tw (Y.-C.C.); cntlichi@nhri.org.tw (N.-T.C.); zeromagi@nhri.org.tw (M.-J.C.); 2 Department of Forestry and Natural Resources, National Chiayi University, Chiayi 60004, Taiwan; kliovemv58@gmail.com; 3 Institute of Environmental and Occupational Health Sciences, National Yang-Ming University, Taipei 11221, Taiwan; wenchipan@ym.edu.tw; 4 Research Center for Environmental Changes, Academia Sinica, Taipei 11529, Taiwan; sclung@gate.sinica.edu.tw; 5 Department of Atmospheric Sciences, National Taiwan University, Taipei 10617, Taiwan; 6 Institute of Environmental Health, School of Public Health, National Taiwan University, Taipei 10055, Taiwan; 7 Institute of Occupational Medicine and Industrial Hygiene, National Taiwan University, Taipei 10055, Taiwan; leonguo@ntu.edu.tw; 8 Department of Geomatics, National Cheng Kung University, Tainan 70101, Taiwan

**Keywords:** land use regression (LUR), ozone, Asian culturally specific source, temple, spatial-temporal variability

## Abstract

This paper developed a land use regression (LUR) model to study the spatial-temporal variability of O_3_ concentrations in Taiwan, which has typical Asian cultural characteristics with diverse local emission sources. The Environmental Protection Agency’s (EPA) data of O_3_ concentrations from 2000 and 2013 were used to develop this model, while observations from 2014 were used as the external data verification to assess model reliability. The distribution of temples, cemeteries, and crematoriums was included for a potential predictor as an Asian culturally specific source for incense and joss money burning. We used stepwise regression for the LUR model development, and applied 10-fold cross-validation and external data for the verification of model reliability. With the overall model R^2^ of 0.74 and a 10-fold cross-validated R^2^ of 0.70, this model presented a mid-high prediction performance level. Moreover, during the stepwise selection procedures, the number of temples, cemeteries, and crematoriums was selected as an important predictor. By using the long-term monitoring data to establish an LUR model with culture specific predictors, this model can better depict O_3_ concentration variation in Asian areas.

## 1. Introduction

A secondary pollutant means something not directly emitted from any source, but formed when primary pollutants react with each other in the atmosphere. Ozone, for example, is a secondary pollutant from the combination of hydrocarbons (e.g., volatile organic compounds (VOCs)) and nitrogen oxides (NO_x_; NO and NO_2_) in the presence of sunlight. Elevated O_3_ concentrations impact air quality and have become a serious environmental concern in Taiwan. In particular, Taiwan’s O_3_ concentration can easily reach 100 μg/m^3^, an upper limit in the Air Quality Guidelines set by the World Health Organization (WHO) in 2005. A previous study [1] and data from the Environmental Protection Agency (EPA) (https://taqm.epa.gov.tw/taqm/tw/default.aspx) also showed that ambient O_3_ concentrations are still increasing in Taiwan. As a strong oxidant, O_3_ causes materials to age rapidly and is toxic to plants [2]; in addition, O_3_ irritates human respiratory systems. Epidemiological studies have confirmed associations between O_3_ and hospital admissions or emergency visits for diminished lung function, respiratory conditions, and other various health outcomes [3,4]. Controlled human exposure studies also showed significant changes in Forced Expiratory Volume (FEV1), respiratory symptoms, and airway inflammation [5,6,7,8]. That said, it is still relatively unknown how long-term ozone exposure may impact human health [9]. Thus more epidemiological studies on ozone exposure are needed. When it comes to the study of health impacts by pollution exposure, the spatial variability of pollution concentration is essential. Some modeling has been used to simulate pollution concentrations, such as the inversion of satellite remote sensing images, interpolation (e.g., inverse distance weighing and kriging), chemical transport models, and Bayesian maximum entropy methods [10,11,12,13,14,15,16]. However, these methods are designed to simulate large geographical areas and thus do not provide a fine-scale variability which is fundamental for understanding ozone exposure [9]. On the other hand, land use regression (LUR) models can better estimate the fine spatial variability of outdoor air pollution and have been widely used in the past decade [17,18,19,20]. However, few have been developed for ozone exposure [21,22,23,24].

This study aimed to develop an LUR model for O_3_ concentration based on 15 years of O_3_ concentration data at 73 EPA automatic monitoring stations across Taiwan. In addition, we also used the Normalized Difference Vegetation Index (NDVI) and the number of temples as variables in developing the LUR model, which are rarely used but important variables in Taiwan. This study provides particularly useful information when developing LUR models in other Asian cities. In terms of local residents’ health outcome or health effect indicators, this study offers much-needed support for air epidemiological studies of O_3_ in the future.

## 2. Materials and Methods

### 2.1. Study Area

Taiwan is an island country in Southeast Asia with China to the west, Japan to the northeast, and the Philippines to the south. Taiwan has 14 counties and 368 townships, covering a geographical area of 36,193 km^2^. With a population of 23,476,640, the average population density is 649 people/km^2^ [25], making it the 17th most heavily populated country in the world. Notably, there are 22 million registered motor vehicles (including motorbikes, cars, and other vehicles) on this small island, or 91.5 vehicles per hundred people [26]. As a result, traffic emissions are a significant factor in urban air pollution [27]. Moreover, on average there are 2.31 factories per square kilometer and many of them are located near commercial districts and residential areas [28]. Local culture also plays a role in this study since there are unique emission sources of O_3_ precursors in Taiwan, such as the frequent burning of joss paper and incense in thousands of temples [29,30]. These two main emission sources not only elevate the level of pollutants but also increase the difficulty in predicting the spatial-temporal variability of O_3_ in Taiwan.

### 2.2. Air Pollutant Database

Taiwan’s EPA has established 73 air quality stations island-wide to systematically monitor daily O_3_ concentrations, including 56 general stations, five traffic stations, four industrial stations, two national park stations, four background stations, and two “other” type stations. General stations monitor the ambient air condition for general residential areas, while the other types of stations take measurements in their respective areas. The two “other” type stations are used to study air pollutants under terrain effects near the Central Mountain Range, which runs north–south along the island. EPA data from 2000 and 2013 were used to develop the model, while observations from 2014 were used as the external data verification to assess model reliability.

These daily measurements were aggregated into annual and monthly averages for model development, resulting in a total of 939 and 10,660 valid measurements, respectively. Concentrations of NO_x_, the precursor of O_3_, were obtained from the EPA database as well and used as explanatory variables since previous studies have confirmed its association with O_3_ concentration [21,31].

### 2.3. Geo-Spatial Database

**Figure 1:**
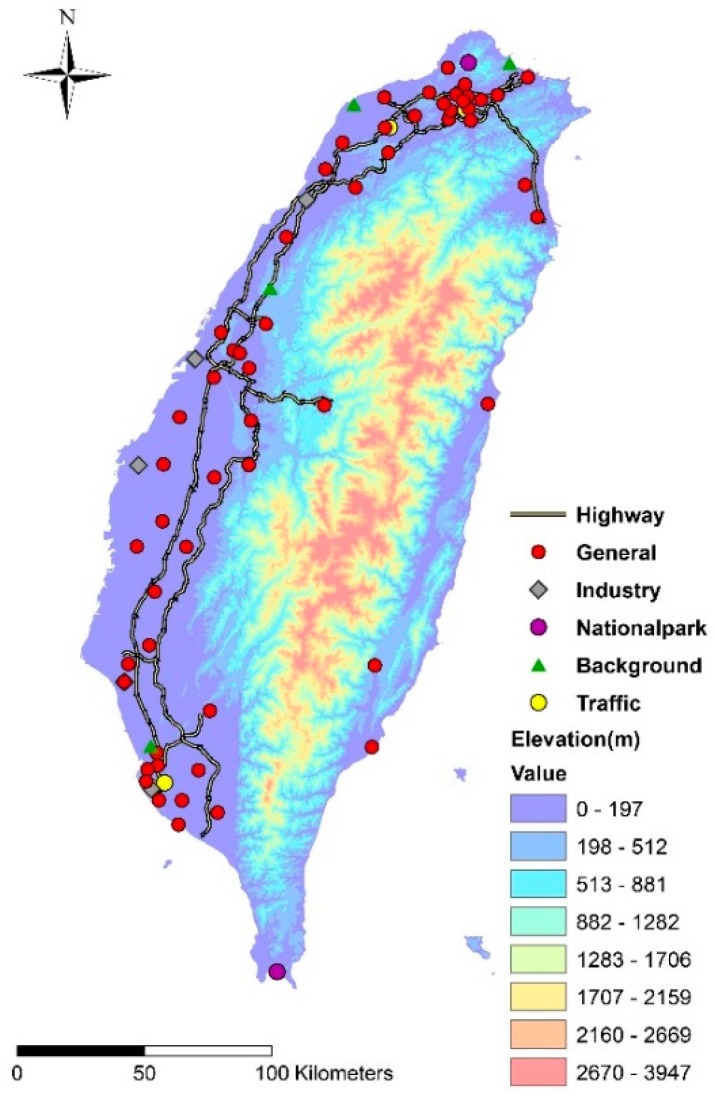
Annual average O_3_ levels at six types of monitoring stations over the study years.

Several Geographic Information Systems (GIS) maps/databases were used to derive land use/land cover variables for the LUR model development, including purely residential, mixed residential, farm, forest, park, water, airport, and port areas from the National Land Use Inventory of 2006 and 2012; industrial parks from the 2010 digital map of industrial parks; road patterns from the digital road network map; and topographic altitudes of the EPA monitoring sites from the Digital Terrain Model with 20 m resolution. Taiwan has 11,275 officially registered temples (and many others unregistered). On average, each county or city in Taiwan has 451 temples. Previous studies suggested that joss money and incense burning would emit the precursor of ozone (e.g., NO_x_ and VOCs) [32,33,34]. We thus also used the number of temples, cemeteries, and crematoriums as well as their locations to collect data from the landmark databases of 2006 and 2008 for the purpose of this study. This EPA database includes more than 0.25 million landmark points in Taiwan. This database is also used in Google Earth to characterize the landmark distribution in Taiwan [19]. The distances to the nearest power plant and garbage incinerator were also calculated and incorporated in the analysis. Moreover, surrounding greenness (e.g., trees and vegetation) from 2006 to 2011 was characterized by NASA’s (National Aeronautics and Space Administration) MODIS (Moderate Resolution Imaging Spectroradiometer) Normalized Difference Vegetation Index (NDVI) database with a 250 m × 250 m spatial resolution. MODIS provides two NDVI measures for each cell every month. In this study, NDVI maps used the acquisition date from mid-month (the fifteenth). All of these geo-spatial variables are abstracted from 25 m to 5000 m circular buffer ranges surrounding each air quality monitoring site, and measured every 25 m, to represent the neighborhood land use/land cover allocations. Figure 1 shows the spatial distribution of air quality monitoring stations in Taiwan, and Table 1 lists potential predictor variables and data sources used in this study.

### 2.4. LUR Model Development and Validation

We built the land use regression model following a methodology developed in our previously published paper [19]. Basically, a supervised stepwise procedure was used to maximize the percentage of explained variability (R^2^). For all potential predictor variables, we chose an a priori direction of effect on O_3_ concentration (e.g., negative for road length and residential area, and positive for NDVI and green spaces) [21,22]. The model started with the variable having not only the highest explained variance in a univariate analysis but also a regression slope with the expected direction. Then all other variables were added to this model separately by assessing if the *p*-value was <0.1 and the variance inflation factor (VIF) was <3. This procedure continued until none of the variables could fit the criteria mentioned above. Finally, we used R^2^, adjusted R^2^, and Root Mean Square Error (RMSE) to assess the model’s performance.

To validate the reliability and robustness of the developed LUR models, two methodologies were used in this study. We first implemented a 10-fold cross-validation methodology to assess the model’s performance [21]. We used 90% of the measurement data from the air quality monitoring sites and the corresponding data of collected variables to develop the LUR model. When the model was developed, we then estimated the annual O_3_ concentration by setting the annual data of each variable. Then, we compared the estimated O_3_ concentrations to the remaining 10% of the measurement data from the air quality monitoring sites. After repeating the same procedure 10 times, each monitoring site served as a validation benchmark at least once. The R^2^, adjusted R^2^, and RMSE values were recorded to evaluate the goodness of fit and robustness of the model. In the second methodology, we used the 2014 data as the validation data and the remaining observations as the training data for model development to assess the accuracy of the external verification.

## 3. Results

### 3.1. Descriptive Statistics of O_3_ Concentrations

**Figure 2:**
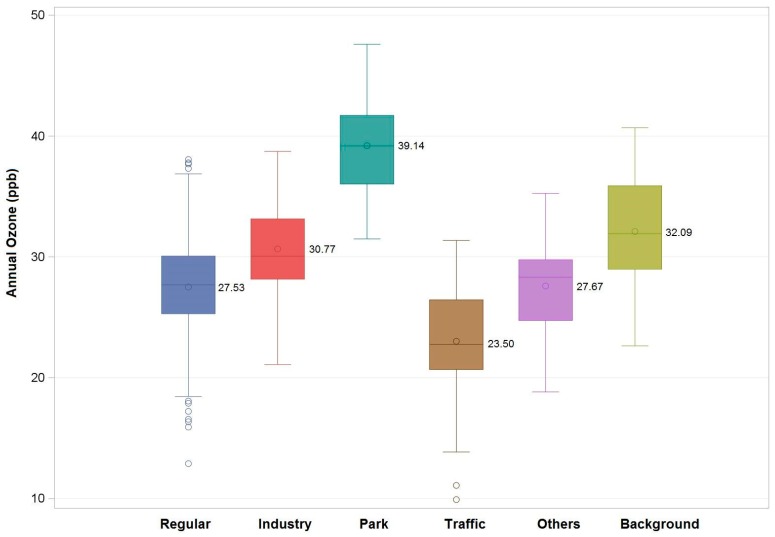
Annual average O_3_ levels at six types of monitoring stations over the study years.

Overall, the level of ozone did not change statistically during the 16-year period (*p*-value <0.01). The annual mean concentration of O_3_ for all monitoring sites in Taiwan was 27.96 ± 3.98 ppb (54.89 ± 7.81 μg/m^3^), which is higher than those in the Netherlands (35.80 ± 5.50 μg/m^3^), Augsburg, Germany (38.20 ± 3.10 μg/m^3^), and Nanjing, China (48.50 ± 3.88 mg/m^3^) [21,22,23], but lower than Linan, China (82.06 μg/m^3^) [35]. Figure 2 shows the annual average O_3_ levels at six types of monitoring stations over the study years. The highest level of O_3_ is 39.14 ppb at a national park station, followed by 32.09 ppb at a background station, 30.77 ppb at an industry station, 27.67 ppb at an “other” type station, 27.53 ppb at a regular station, and 23.50 ppb at a traffic station. Generally, there are lower concentrations in urban areas, especially at traffic sites.

### 3.2. LUR Model Assessment

Table 2 shows the coefficient estimate, partial R^2^, and overall performance of the LUR model developed in this study. With the overall model R^2^ of 0.74 and an averaged, 10-fold cross-validated R^2^ of 0.70, this model presents a mid-high prediction performance level. Even when the external data validated with the R^2^ value was reduced to 0.39, this model still showed a mid-range prediction performance level. The major variables selected are statistically significant predictors for the developed models including concentrations of NO_x_, distance to thermal power plants, all types of residential areas within 25 m, relative humidity, forest within 500 m, altitude, distance to main road, purely residential areas within 25 m, cemeteries and crematoriums within 3000 m, temples within 500 m, temperature, non-irrigated crops within 250 m, temples within 1000 m, and industrial areas within 5000 m. Most variables show a negative association with O_3_ except for forest, altitude, and non-irrigated crops. Temples, cemeteries, and crematoriums were collected in the final model, indicating that the pollutant level was affected not only by well-known pollution sources but also by unique local sources. In our model, the NO_x_ concentration was first entered into the model with a partial R^2^ = −0.54. The highly negative correlation with NO_x_ was lower than that in the Netherlands (R = −0.87) [21] but higher than that in Neuherberg, Germany (R = −0.32) [22], which suggests a substantial photochemical effect on ozone–NO_x_ associations in Taiwan [22]. Such negative correlation between O_3_ and NO_x_ also presents a challenge to minimize the health impact by ozone and pollutants from primary factories, thermal power plants, and traffic emissions. Taiwan, while being a small island, has the highest motor vehicle density in Asia with an average of 378 vehicles per square kilometer. Likewise, Taiwan has the highest number of scooters per square kilometer in the world. Indeed, the vehicle-emitted NO_x_ [21] was selected as the prediction variable with the highest partial R in our LUR model. We can thus conclude that emission by motor vehicles is the most dominant factor that affects the O_3_ concentration in Taiwan. The high population density in Taiwan (649 person/km^2^) might also have an impact on O_3_ concentration since all types of residential areas were selected in our model. Moreover, a highly negative correlation with relative humidity was obtained. The underlying reasons for the reduction of O_3_ in the presence of water vapor are not clear, although it is known that the water vapor present in the air affects the corona initiation field strength [36,37]. In addition, we found a positive correlation between O_3_ and forest within a large buffer, consistent with a previous study [21]. The forest predictor suggests the absence of primary NO_x_ sources and/or biogenic VOCs, either of which may increase ozone formation.

### 3.3. Spatiotemporal Variations of O_3_

**Figure 3:**
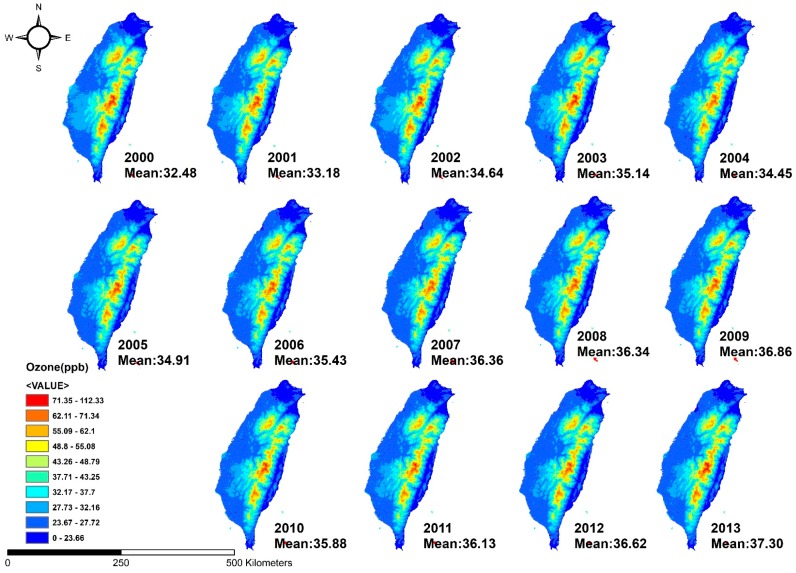
Annual average O_3_ concentration for the entire study period as simulated by the developed model.

Figure 3 illustrates the annual average O_3_ concentration for the entire study period, as simulated by the developed model. Red to blue on the maps represents the levels of ozone pollution from high to low. Eastern areas clearly have higher O_3_ concentrations throughout the predicted period. The highs and lows of O_3_ concentrations in different areas of Taiwan is consistent with existing knowledge about ozone formation and destruction. For instance, relatively high O_3_ concentrations are observed in highland areas in many regions of the country [38,39,40]. In this study, O_3_ concentrations close to the Central Mountain Range are also higher than those on the plains. However, the O_3_ concentrations on the top of the mountain should not be used because of the lack of monitoring stations in this area.

## 4. Discussion

While land use regression models have become increasingly popular for simulating air pollutant concentrations, they are rarely used in Taiwan [19,41]. In addition, although the Taiwan EPA has widely monitored air quality with different characteristics in different areas, there is no proper way to use the large-scale monitoring network established by the EPA to estimate public exposure. Thus, this paper used LUR models to estimate O_3_ concentrations across the island according to the EPA’s monitoring network. This study shows a successful combination of LUR models for air pollutants and the EPA’s publicly available data from its national monitoring network. This is also significant for the epidemiological studies which need data with fine-scale exposure concentrations.

The descriptive statistics of on-site O_3_ observations show lower concentrations in urban areas but higher levels in rural areas. This pattern is consistent with the understanding that O_3_ will react with NO emitted by combustion sources including vehicle exhaust [21]. In addition, pollutant sources can impact air quality not only in local areas but also in downwind rural areas [42,43,44,45]. Moreover, the biogenic volatile organic compounds (BVOCs) in the atmosphere of forests are attributed to isoprene and monoterpenes, which have been shown to contribute to the formation of elevated ozone levels.

This could explain why the highest level of O_3_ occurred at a national park station.

In this study, the value of the model R^2^ is 0.74, which demonstrated better model performance than a previous study in Asia, where the model R^2^ was 0.60 [23]. Huang also developed LUR models for O_3_ concentration using data from the Nanjing Environmental Monitoring Center in 2013 but only selected longitude and slope as predictors in the model, both of which are temporally constant. Because of this, Huang’s model cannot be used to predict O_3_ concentration in different seasons or years. In contrast, this study chose temporally variable predictors such as NO_x_ concentration and temperature, meaning we were able to develop a model that is better for estimating O_3_ concentration in different years.

A previous study using an LUR model for O_3_ concentration from Sweden [46] found significantly higher concentrations (60 to 83 μg/m^3^) in Malmö and Umeå (36 to 63 μg/m^3^). However, these mean concentrations of three weekly measurements were conducted in only three months (April, May/June, and August), and it disregarded other months with minimum concentrations. Kerckhoffs et al. [21] developed a national O_3_ model using four bi-weekly programs in each season for the Netherlands and reported much lower annual average O_3_ concentrations, from 25.0 to 47.8 μg/m^3^. In contrast, this research shows a wider range (from 36.73 to 70.18 μg/m^3^) because we covered an area with more diversity to include both remote areas and the metropolitan Taipei, which is four times more populated than Amsterdam. In addition, some of our findings are similar to Kerckhoffs’, wherein higher traffic often leads to lower O_3_ concentrations.

While traffic is often the dominant factor in predicting O_3_ concentrations in the cities of Europe or the United States, some culturally specific O_3_ sources must be considered in Asia. Joss paper and incense burning are very important for many Asian households and temples for religious purposes [47], and several studies have shown their contributions to air pollution [27,48]. Incense and joss money combustion emit VOCs (i.e., benzene, benzo (a) pyrene, and formaldehyde) and NO_x_ [32,33,34,49]. The level of O_3_ is highly correlated with NO_x_ and VOC concentrations in the presence of sunlight [22,50], but none of these studies considered joss paper and incense burning-related variables or developed an LUR model for O_3_ concentration. In this study, we used the number of temples, cemeteries, and crematoriums to reflect local emissions by joss paper and incense burning, which proved to be a significant predictor in our newly developed model. Thus, we suggest that future studies should consider this unique local cultural source as a predictor when establishing LUR models for O_3_ in other Asian regions.

There were some limitations when we selected predictors in this study. For instance, traffic intensity, wind factor, the number of buildings, and population, though used by others to improve model performance [21,22,24], were not used in this study because these data are not readily available in Taiwan. Nonetheless, this model used a tremendous amount of data covering the entire island for the past 14 years to represent spatiotemporal variation of O_3_ concentrations better than previous studies (which only used data for a single year or less and in specific locations). Our LUR model is limited to only provide annual data because of the constant variables (such as residential area, distance to the main road, and number of temples). In addition, the uneven distribution of stations (e.g., few sites located in mountain areas and eastern Taiwan) might lead to some uncertainty. However, the annual data could be used to study long-term health impacts. For instance, in [51,52] the authors used the annual average ozone to evaluate the associations between ozone concentrations and the risk of death. By using long-term monitoring pollutant data to establish an LUR model with culturally specific predictors, this model presents a mid-high prediction performance level, which can be used to better depict O_3_ concentration variation in Asian cities.

## 5. Conclusions

This study is the first to use the spatiotemporal variation of ozone concentration in Taiwan to develop an LUR model. The model shows the spatial variance of ozone over the entire island of Taiwan, especially in the context of ozone being a secondary pollutant. By using data from the EPA’s national monitoring network, which routinely monitors air quality with different characteristics in different areas, and with the consideration of culturally specific predictors such as temples, we confirm that the LUR models developed in this study can predict the fine spatial variability of both long-term and short-term outdoor O_3_ concentrations. Moreover, this LUR method could be similarly used in future studies to develop new LUR models for other pollutants in Taiwan. In terms of local residents’ health or health effect indicators, this study offers much-needed support to air epidemiological studies in the future.

## Figures and Tables

**Table 1:** Potential predictor variables.

Data Source	Variable	Data Description	Unit	Buffer Size (m)
Institute of Transportation digital map data	Road ^a^	Major road	m	25–5000
		Local road		
		All types of road (major road + local road)		
The second national land use survey	Residential Areas	Purely residential area	m^2^	25–5000
		Residential area mixed with industrial area		
		Residential mixed with commercial area		
		Mixed residential area (residential area mixed with industrial and commercial area)		
		All types of residential area (pure and mixed residential area)		
The second national land use survey	Greenness	Paddy rice		
		Non-irrigated crops		
		Fruit orchard		
		Mixed crops (rice + non-irrigated crops + fruit orchard)		
		Forest		
		Park		
The second national land use survey	Industrial area			
The second national land use survey	Water			
Vegetation indices from remote sensing	NDVI		-	250–5000
Point of interest (POI) landmark database	Asian culture-specific emission sources	Temple	count	25–5000
		Chinese restaurant		
		Temple + Chinese restaurant		
		Cemetery and crematorium	m ^a^	NA
The second national land use survey	Port			
The second national land use survey	Airport			
Taiwan Environmental Protection Agency (EPA) environmental database	Incinerator stack			
Taiwan EPA environmental database	Thermal power plant			
Taiwan EPA environmental database	Garbage incinerator			
Taiwan EPA environmental database	Industrial park			
Institute of Transportation digital map data	Main road			
Central Weather Bureau database	Altitude		m ^b^	NA
Taiwan EPA environmental database	Pollutants	CO	ppm	NA
		NO_x_		
Central Weather Bureau database	Meteorological factor	Temperature	℃	NA
		Relative humidity	%	NA
		UV	nm	NA

^a^ distance to the nearest landmark; ^b^ elevation above sea level of the monitoring site.

**Table 2:** Land use regression model for annual average ozone concentration (ppb).

Variable	Regression Coefficient	*p*-Value	Partial R
Intercept	1.52	<0.01	
NO_x_	−4.79 × 10^−3^	<0.01	0.54
Thermal power plant	−1.55 × 10^−6^	<0.01	0.08
All types of residential—25 m	−1.25 × 10^−5^	0.06	0.001
Relative humidity	−1.85 × 10^−3^	<0.01	0.02
Forest—500 m	1.15 × 10^−7^	<0.01	0.02
Altitude	1.03 × 10^−4^	<0.01	0.009
Distance to main road	9.64 × 10^−6^	<0.01	0.005
Purely residential—25 m	−3.25 × 10^−6^	0.13	0.004
Cemetery and crematorium—3000 m	−1.71 × 10^−8^	<0.01	0.004
Temple—500 m	−4.29 × 10^−3^	0.01	0.003
Temperature	9.05 × 10^−3^	<0.01	0.003
Non-irrigated crops—250 m	2.09 × 10^−7^	<0.01	0.002
Temple—1000 m	−4.13 × 10^−4^	0.05	0.001
Mixed residential area—25 m	−9.25 × 10^−4^	<0.01	0.04
Industrial area—5000 m	−1.44 × 10^−9^	<0.01	0.003

Model performance: overall model R^2^ = 0.74; adjusted R^2^ = 0.73; Root Mean Square Error (RMSE) = 0.04 ppb; 10-fold cross-validation R^2^ = 0.70; externally validated R^2^ = 0.39.
